# Drug Target Protein-Protein Interaction Networks: A Systematic Perspective

**DOI:** 10.1155/2017/1289259

**Published:** 2017-06-11

**Authors:** Yanghe Feng, Qi Wang, Tengjiao Wang

**Affiliations:** ^1^Science and Technology on Information Systems Engineering Laboratory, National University of Defense Technology, Changsha 410073, China; ^2^Department of Bioinformatics, Second Military Medical University, Shanghai, China

## Abstract

The identification and validation of drug targets are crucial in biomedical research and many studies have been conducted on analyzing drug target features for getting a better understanding on principles of their mechanisms. But most of them are based on either strong biological hypotheses or the chemical and physical properties of those targets separately. In this paper, we investigated three main ways to understand the functional biomolecules based on the topological features of drug targets. There are no significant differences between targets and common proteins in the protein-protein interactions network, indicating the drug targets are neither hub proteins which are dominant nor the bridge proteins. According to some special topological structures of the drug targets, there are significant differences between known targets and other proteins. Furthermore, the drug targets mainly belong to three typical communities based on their modularity. These topological features are helpful to understand how the drug targets work in the PPI network. Particularly, it is an alternative way to predict potential targets or extract nontargets to test a new drug target efficiently and economically. By this way, a drug target's homologue set containing 102 potential target proteins is predicted in the paper.

## 1. Introduction

Drug target studies have been conducted in both dry and wet labs from experimental designs to target identification and validation steps. There are two ways for target discovery: a system approach and a molecular approach [1, 2]. In 2006, Imming et al. [3] catalogued 218 molecular targets for approved drug substances and Overington et al. [4] proposed a consensus number of 324 drug targets for all classes of approved therapeutic drugs. Rask-Andersen et al. [5] analyzed trends in the introduction of drugs that modulate previously unexploited targets and discussed the network pharmacology of the drugs in our dataset. Yildirim et al. [6] built a bipartite graph composed of US Food and Drug Administration-approved drugs and proteins linked by drug target binary associations. By using chemical 2D structural similarity, Keiser et al. predicted new molecular targets for known drugs [7]. Campillos et al. [8] used phenotypic side-effect similarities to infer whether two drugs share a target. The main molecular targets for drugs are proteins (mainly enzymes, receptors, and transport proteins) and nucleic acid (DNA and RNA). It is indeed gratifying that development of research in cell biology, molecular biology, and biochemistry produced a remarkable compendium of knowledge on the function and molecular properties of individual proteins [9]. However, a protein usually carries out a typical function by regulating other molecules. In other words, it rarely acts alone. Therefore, in the past few years, with the emergence of high-throughput technologies for omics data, like yeast 2 hybrid protein interactions, research of protein-protein interactions (PPI) has been occurring with high frequency. Bultinck et al. [10] concluded that a growing number of functional PPI modulators are being reported and clinically evaluated. PPI provides us with more information for understanding the relationship between drug targets and other proteins in a systematic point of view.

Many researchers believe that topographical analysis of the complex network of intercellular protein interactions may also lead to new avenues for target prediction [11]. Based on the graph modeling theory in computer science, protein networks are extensively being studied in the system biology field. Royer et al. [12] used power graph analysis to explicitly represent reoccurring network motifs. In a systematic point of view, the gene regulatory network and drug targeted protein network are different from their normal counterparts. Currently, the interactions between drugs and targets have also been studied as an interactive network. Chautard et al. [13] described the high-throughput methods used to identify new interactions and to build large datasets that record the identified interactions. Yamanishi et al. [14] characterized four classes of drug–target interaction networks and revealed significant correlations between drug structure similarity, target sequence similarity, and the drug–target interaction network's topology. The essential intentions of these approaches are trying to take the time-specific or space-specific information into account. Thus, these systems biology approaches will lead to time-sensitive, space-sensitive, and synergistic treatments taking the multidimensional use of drugs into consideration [13].

These studies mainly focused on interactions of drugs targets rather than the targets' topological features. It is a reasonable way to identify targets on which the typical drugs work. However, the targets topological features are helpful to predict new targets because most of them have similarity on some topological features which are different from normal proteins. To construct a panoramic view of drug targets, in this paper, we examined the three main institutive views about drug target characteristics: intermediaries, source of the drug stimulus, and special topological features. Based on the PPI network, we analyzed lists of the topological indices related to the three traditional views above. The results show, somewhat surprisingly, that the topology of a drug target is not to help it as intermediary or be the source of the drug stimulus. On the other hand, drug target proteins indeed have some special topological features that are significantly different than normal proteins.

The remainder of this paper is organized as follows. The Data Collection elaborates the details about data sources of drug target properties and the PPI network used in the paper. The analysis on drug target protein topology under three transitional points is in the Analysis. The Discussion discusses the drug target's topological characteristic and its applications. This paper concludes in the Conclusion with the impact of the paper and our future work insights.

## 2. Data Collection

Proteins are the main catalysts, structural elements, signaling messengers, and molecular machines of biological tissues [15]. The interactions between proteins form the basis for signal transduction pathways and transcriptional regulatory networks. Therefore, it is very important in harmonizing the events in a cell. Target proteins are functional biomolecules that are addressed and controlled by biologically active compounds. Currently, the main resource of protein interactions is from five most widely used PPI databases (HPRD version R9) [16], IntAct (2010-02-17 downloaded) [17], BioGRID (version 2.0.63) [18], MINT (version 2010-05-05) [19], and DIP (version 2009-12-30) [20]. The databases contained a combined 65,785 nonredundant interactions.

In this paper, the main information of drug targets was extracted from the DrugBank database, in which the approved targets set (version 3.0) [21] contains 1,604 proteins. Then PISCES [22] was used to remove those sequences with an identity larger than 20% for both the drug target and the nontarget sequence. Using this method, we gained 517 drug targets and 3,834 common proteins. The drug protein properties included single peptide cleavage [23], transmembrane helices [24], low complexity region [25], N-glycosylation [26], and O-glycosylation [27]. These chemical properties of the amino acids of proteins determine the biological activity of the protein. Therefore, they can provide us with information that whether a protein is suitable to be a drug target protein. The chemical and physical properties which were used in our analysis are 26 amino acids (counted by Mole%) and number of charged residues, basic residues, acidic average molecular weight, and isoelectric point are used to train the model and predict the potential drug targets [28]. Besides, they are important clues as well as the PPI topological features for the judgement of which proteins could be targets. Here we use pepstats, an online software from EMBOSS [29] to calculate statistics of protein properties. A more detailed information on these properties including hypothesis test is in [30]. Finally, the proteins dataset contains 39 chemical and physical properties.

After integrating the DrugBank target protein data and the PPI data, we gained 1,361 proteins that have both network properties and chemical-physical properties. Of the 1,361 proteins, 149 are known drug targets and the remaining 1,212 are yet to be tested. There are 10,197 proteins without chemical and physical properties. Unfortunately, although there are improved methods to reduce the protein-protein interaction's false positive rate [31, 32], the information of protein-protein interactions is always incomplete so that there are some isolated nodes (e.g., PDXP, ODZ1, NT5M, and IL17F) and small components that only contained two or three nodes (e.g., the component consisting of KLRG1 and LEPROTL1 and the component consisting of PCYT2 and JMJD5). Hereby, in order to reduce the effect from lack of complete interactions, we used the maximal connected component instead of the original PPI. It contains two types of proteins, 138 drug targets (D) and 11,163 pending test proteins (PT). The latter consist of 1,180 proteins with 39 chemical-physical properties (PT1) and 9,983 without any properties (PT2).

Table 1 shows the overall description of the collected data. It gives a summary of the data which were performed in our manuscript. It shows the fact that the number of known drug target proteins is far less than the number of pending test proteins. It reveals the feasibility of this analysis and the possibility that there are many potential drug targets in the pending test data. The known DT proteins which we collected from the DrugBank database were removed from non-DT proteins dataset. The proteins redundancy method [33] was performed to obtain our DT and non-DT protein dataset. The coverage ratio is calculated in the way using the used data (the number of drug target or pending test proteins which is used in the analysis) divided by the original data (the number of drug target or pending test proteins acquired from the public dataset).

## 3. Drug Target Network Topology Analysis

As stated in classical complex networks theory, the drug targets network is represented as an undirected network *G* = (*V*, *E*), where *V* denotes the protein in D set or PT set and *E* is the interactions between each proteins pair. In the paper, the drug targets network contains 11,301 nodes and 65,547 edges. For each node *i* ∈ *V*, *k*_*i*_ denotes the degree of it. *A* is the adjacency matrix for the network, where  *A*_*i*,*j*_ = 0 when there are no interactions (no edge) between nodes *i* and *j*. Similarly, *A*_*i*,*j*_ = 1 when there is an interaction between each other.

The drug target is the native protein in the body whose activity is modified by a drug resulting in a desirable therapeutic effect. Different drugs act on molecular targets at different locations in the cell. In the human body, all cells have membrane that enclose the cytoplasm. The cell membrane consists of two identifiable layers, each of which is made up of an ordered row of phosphoglyceride molecules such as phosphatidylcholine. A phosphoglyceride molecule consists of a small polar head group and two long hydrophobic chains. In the cytoplasm, there are several structures, one of which is the nucleus that acts as the control center for the cell. There are also many other structures within a cell, such as mitochondria, Golgi apparatus, and the endoplasmic reticulum. As the drug targets are the special proteins through which the drugs carry out their specific functions, they are thought institutively as (1) the intermediaries which play an important role on interactions of the drug targets network; (2) the sources which receive the drug stimulus and convert it into another stimulus that can be responded to by normal proteins; (3) the proteins which have special topological and functional significance. According to these three points, we analyzed the listed topological features of the drug targets network, including degree and betweenness for the intermediary function, eccentricity, and average distance for the source function, modularity, coreness, cluster coefficient, and eigenvector centrality for special topology. However, from analyses of the PPI topological indices, the drug targets do not have the first two characteristics. Actually, the results show they are similar with other proteins on intermediary and source functions. In comparison, there are some significant differences on special topology.

### 3.1. Drug Target as Intermediary

Diseases are regulated by complex biological networks and depend on multiple steps of genetic and environmental challenges to progress [34]. Disease-relevant intracellular PPI occurring at defined cellular sites possess great potential as drug targets. They permit highly specific pharmacological interference with defined cellular functions [35]. In other words, the drug targets seem to be the proteins through which the drug effects tend to be spread over the PPI to stimulate other related proteins. In this paper, we studied the degree and betweenness to analyze the intermediary function of the drug targets.

#### 3.1.1. Degree

**Figure 1:**
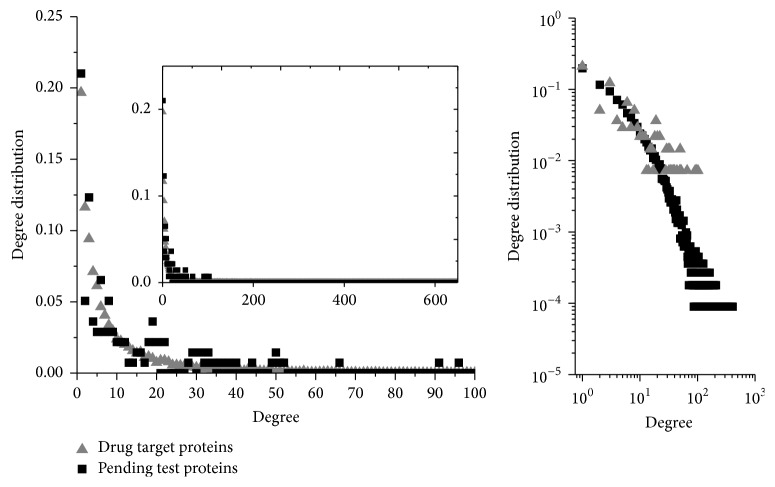
Degree distributions of two types of proteins. The inset is the overall view of both distributions. Drug targets and common proteins are following the well-known power law just with different parameters. The highest degree of the drug targets is 103 compared to 667 in PT. It indicates that the drug targets are not the hubs.

The degree of a node in a network (sometimes referred to incorrectly as the connectivity) is the number of connections or edges the node has to other nodes. In protein interaction networks, the hubs are defined as ones that have a higher degree than others; for example, Vallabhajosyula et al. [36] suggested that some of the highest degree proteins should be defined as hubs which have special topological and functional significance. We analyzed the degree distribution of the known drug targets and other proteins from Pending Proteins (PT) shown in Figure 1, where the *x*-axis means the degree and the *y*-axis means the proportion of the protein with that degree. The right subfigure, the double logarithm coordinate system, shows an obvious power-law distribution with significant long tail characteristics for both groups. The highest degree of the drug targets is 103 compared to 667 in PT. The average of degree in drug targets is 13.3 compared to 11.6 of the proteins in PT.

Figure 1 shows the degree distributions of two types of proteins during *k*_*i*_ ∈ [1,103] in which all of the known drug targets distribute. The inset is the overall view of both distributions. From Figure 1, the proteins with the highest degree in the drug targets network are usually not drug targets. On the contrary, the degree distributions are similar between drug targets and the majority of other proteins. They are following the well-known power law just with different parameters, which seems to be counterintuitive. The result implies that the drug target proteins are actually not “hub” proteins but rather they probably carry specific functions at a certain level of interactome as a common protein. Due to robustness and resilience of the PPI network based power law, the topological properties will not change if some small degree nodes are removed. But for the hubs, Vallabhajosyula et al. [36] referred that removement of the hubs would cause significant change for the PPI network. According to it, we analyze the change of degree after the drug targets are removed. There is only decreasing from 11.6 to 11.4 after those drug targets were removed. Therefore, the results imply that the degree distribution of the drug targets is not a significant topological network feature. Actually, it is similar to cancer research findings such as the findings of Barillot et al. [37] which propose a notion about a router that is not necessary to have a high connection like a hub. However, it always plays an important role of propagating the biological signal to a local hub or a global hub protein.

#### 3.1.2. Betweenness

Betweenness is the number of times a node is in the shortest paths between two other nodes. In the PPI network, if the drug targets are the proteins that play an important role on intermediary, they may have higher betweenness than others. Actually, many studies show that some interactions are more important in nonhub proteins based on betweenness. For example, in the yeast proteins network, the coordinated functionality is carried out by the connectors which have high betweenness, even though they have low degree [38]. Particularly, in the PPI network which has undirected edges, Yu et al. [39] found the betweenness is more essential than degree with gene essentiality and expression dynamics. Therefore, we try to study the intermediary function of the drug targets based on betweenness.

The betweenness centrality of a node *v* is given by

(1) $$\begin{array}{c} Btwn\left(\;v\;\right)=\underset{s\neq v\neq t}{\sum }\frac{\sigma_{st}\left(v\right)}{\sigma_{st}}, \end{array}$$

where *σ*_*st*_ is the total number of shortest paths from node *s* to node *t* and *σ*_*st*_(*v*) is the number of those paths that pass through *v*.

The normalized betweenness (NB) can be calculated without a loss of precision

(2) $$\begin{array}{c} NB\left(\;v\;\right)=\frac{Btwn\left(v\right)-\min\left(Btwn\right)}{\max\left(Btwn\right)-\min\left(Btwn\right)}. \end{array}$$

**Figure 2:**
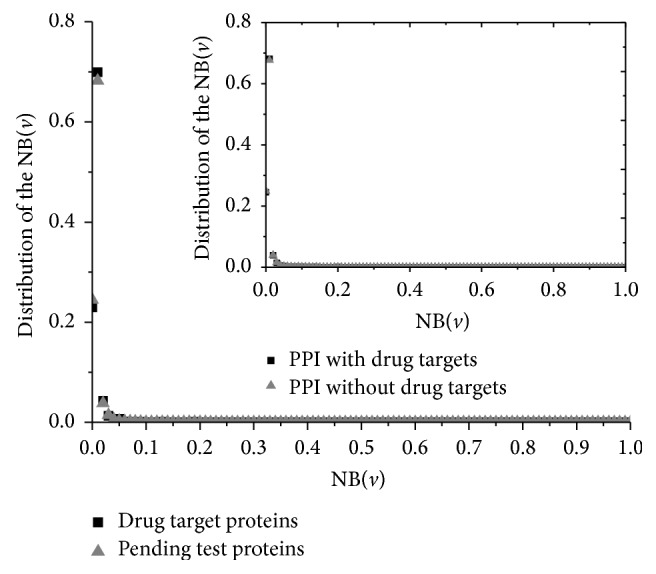
Distribution of normalized betweenness of drug targets proteins and pending test proteins. The betweenness of drug target proteins is lower than others. The distributions of the two types of proteins are similar. The inset shows there are few changes between the betweenness distributions of the original PPI and the new PPI with the known drug targets removed.

It results in max⁡(NB) = 1 and min⁡(NB) = 1.  Figure 2 showed the distributions of the drug targets and other proteins in the PPI network.

In Figure 2, there is a surprising result as the conclusion from analysis of the degree. The drug target proteins do not have high betweenness but rather lower than most common proteins. The highest NB of the drug target proteins is BCL2_HUMAN which is only 0.0497. Even though the drug targets are all removed from the PPI network, the betweenness of the remaining proteins is nearly unchanged shown in inset of Figure 2. It also clearly shows that the drug target proteins hold the similar distribution of betweenness as other proteins. This implies that relevance of a drug protein as an organizing regulatory molecule is fairly the same as other proteins.

**Figure 3:**
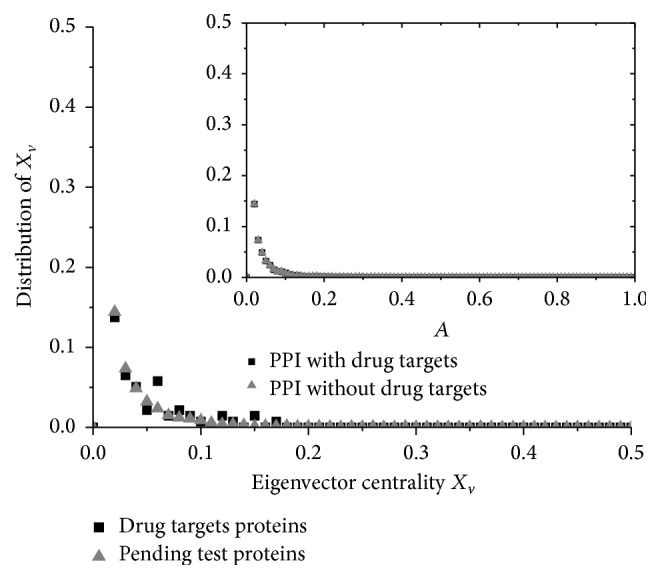
Distribution of eigenvector centrality. Drug targets and other proteins have the same distribution of the eigenvector centrality. The inset shows that the amount of changes on the eigenvector centrality after removing the drug targets is negligible. But the average eigenvector centrality of drug targets is not lower than others although the results of the analysis on degree and betweenness implied the drug targets are not the hubs.

From the analysis on degree and betweenness, it implies that the drug targets are not the important proteins from the view of the connectivity of the PPI network. In a complex network, the eigenvector centrality is a measure of the influence of a node. It assigns relative scores to all nodes in the network based on the concept that connections to high-scoring nodes contribute more to the score of the node in question than equal connections to low-scoring nodes [40, 41]. We examined whether the eigenvector centrality *x*_*v*_ of the drug target is lower and the distribution is similar to the normal proteins (Figure 3). The centrality score *x*_*v*_ of vertex *v* is defined as

(3) $$\begin{array}{c} X_{v_{i}}=\frac{1}{\lambda }\underset{v_{t}\in M\left(v_{i}\right)}{\sum }X_{v_{t}}=\frac{1}{\lambda }\underset{V_{t}\in G}{\sum }A\left(\;v_{i},v_{t}\;\right)X_{v_{t}}, \end{array}$$

where *M*(*v*_*i*_) is a set of the neighbors of *v*_*i*_ and *λ* is the greatest eigenvalue of the adjacent matrix *A*.

Eigenvector centrality measures the centrality of a node by judging how many nodes it connected to with high degree. The distributions of the drug target proteins and other proteins are quite similar as shown in Figure 3. Even if the drug targets are removed, the change of the eigenvector centrality is so small that it can be ignored (see inset of Figure 3). It seems to be the same as what degree and betweenness imply. However, the average eigenvector centrality of drug targets is 0.022, which is not lower than normal proteins 0.02. This conclusion seems to oppose the conclusion that drug targets are not the important proteins from the view of the connectivity based on the degree and betweenness. It indicates that the drug targets have interactions with the high degree proteins although they are not hubs of the network as what degree and betweenness implied. Actually, it is the important feature of the drug targets and is analyzed again when we consider coreness of the PPI network.

Intuitively, the drug effects are spread by some special proteins called drug targets which have either high degree or high betweenness. Actually, in many studies on protein interactions or gene interactions, the hubs or the nodes with high betweenness are the important ones [42–44]. However, the conclusions of our analysis are opposite: the drug targets have lower degree and betweenness, but also there are no significant differences on the distributions of both topological indices between drug targets and other proteins. It implies that the intermediary is not the main function of the drug targets. There are many potential reasons for this. One reason is that the drug design method is based on the ligand and structure at present. Therefore, the drug's stimulus is effective for some specific proteins rather than a large amount of proteins.

### 3.2. Drug Targets as Source

The drug actions depend on the complex signaling transduction networks of cells or the complicated profile of drug potency and selectivity [45]. In most cases, if the different targets stimulated by a drug, the in vivo effect on the signaling pathway should be changed and the drug's efficiency to inhibit the activity (usually measured as phosphorylation level) will be different [46]. In other words, it is possible that a drug target seems to be the source which receives the drug stimulus and converts it into another stimulus that can be responded to by normal proteins. If so, the drug targets should be the sources from which it is convenient to access other proteins in PPI networks. Even if a protein has low degree and betweenness, it is possible for it to have short distances with others. More generally speaking, the convenience of the drug targets depends on the degree and betweenness analyzed above but also the distances to other proteins. In this paper, we studied the average distance and eccentricity to analyze source functions of drug targets.

#### 3.2.1. Average Distance

**Figure 4:**
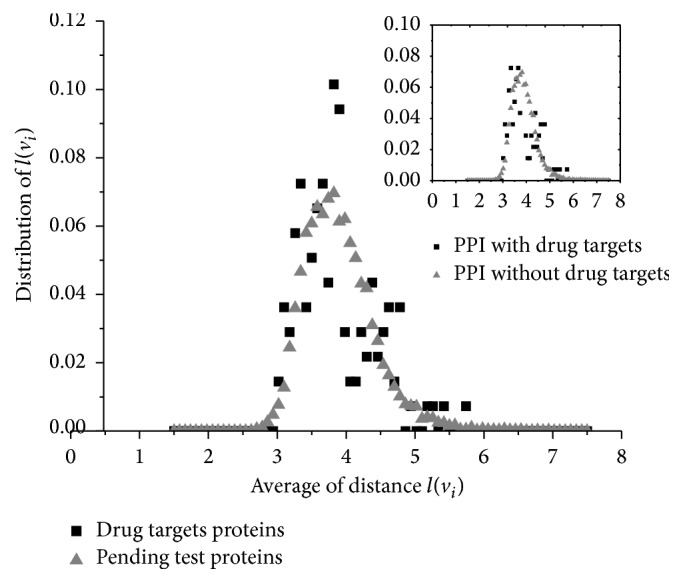
Distribution of average distance. The known drug target's distribution of average distance is similar with common proteins. The inset shows there are few changes on the average distance after the protein targets were removed from the PPI network.

Average distance, also called average path length, is a concept in network topology that is defined as the average number of steps along the shortest paths for all possible pairs of network nodes. Here, we consider an unweighted graph *G*; let *d*(*v*_*i*_, *v*_*j*_) denote the shortest distance between *v*_*i*_ and *v*_*j*_. Assume that *d*(*v*_*i*_, *v*_*j*_) = 0 if *i* = *j* or *D*_*G*_ if *v*_*i*_ cannot be reached from *v*_*j*_, where *D*_*G*_ is the diameter of the PPI network. Note that it is different with the traditional average distance which defines *d*(*v*_*i*_, *v*_*j*_) = 0 if *v*_*i*_ cannot be reached from *v*_*j*_. However, we are concerned about how a protein spreads the effects of the drug to others. If there are no interactions between two proteins, the stimulus on each drug is not transferred to the other. The distribution of the average distance *l*(*v*_*i*_) is shown in Figure 4.

The average distance shows the overall convenience of the proteins to communicate and/or affect their reciprocal function. It is also a sign of functional convergence [47]. In PPI networks, the special proteins with low average distance cause many diseases. For example, when the cancer related proteins are compared with normal proteins, the average distances are lower [42]. But in our study, the drug targets do not have this feature. Figure 4 shows that the distribution of average distance is similar with other proteins. Meanwhile, even if the protein targets are removed from the PPI network, the distribution of average distance hardly changed. Hence, the drug targets stimulated by a special drug do not have more significant convenience of propagating the effects to other proteins.

#### 3.2.2. Eccentricity

**Figure 5:**
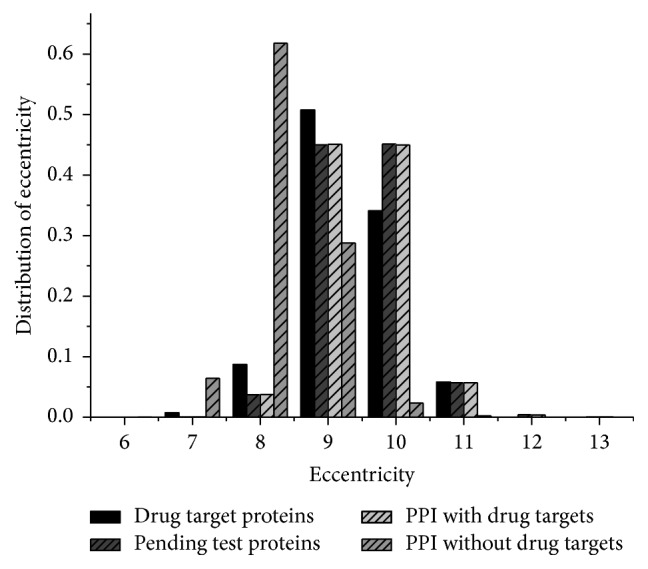
The distribution of eccentricity. It shows that the difference in eccentricity between drug targets and others is not remarkable although most targets' eccentricity is 9 and the normal proteins' eccentricity is 9 and 10. However, the eccentricity is changed apparently after removing the drug targets from PPI networks.

The eccentricity *ϵ*(*v*) of a vertex *v* is the greatest geodesic distance between it and any other vertex [48]. It can be thought of as how far a protein is from the proteins farthest from it in the graph [49]. Indeed, if the eccentricity of the node *v* is low, the other nodes are in proximity. On the contrary, if it is high, it implies that there is at least one node (and all its neighbors) that is far from node *v*. Let *d*(*v*_*i*_, *v*_*j*_) denote the distance (number of edges connected) between vertices *i* and *j*; then the eccentricity *ϵ*(*v*) = max_*v*_*i*_,*v*_*j*_∈*V*_⁡*d*(*v*_*i*_, *v*_*j*_).  Figure 5 shows the distribution of the eccentricity.

The eccentricity shows the easiness of a protein to be functionally reached by all other proteins in the network. Thus, a protein with low eccentricity is subject to a more stringent or complex regulation so that it could easily influence several other proteins. Similar to the average distance, there are few observed differences on the distribution of eccentricity between known drug targets and other proteins (Figure 5). However, after removing the drug target proteins from the original proteins network, the eccentricity of most proteins reduced to 8 compared with 9 and 10 in the network with drug targets (Figure 5). In a network, the eccentricity *ϵ*(*v*) should be increased if the hubs or some important connectors are removed. Hence, the results above including analysis of the intermediary showed, somewhat surprisingly, that the drug targets are not the proteins that play the important role on connectivity of the PPI networks. On the contrary, most of them are stimulated by drugs and this modification on their activities is spread to other proteins. Therefore we hypothesized that the drug mainly fulfills effects on some special targets, rather than stimulating other proteins through drug targets. For example, if avoiding the host's defense mechanisms and inhibiting nonspecific distributions in the liver and spleen by targeted drug delivery, a cardiac tissue system can reach the intended site of action in higher concentrations [50].

### 3.3. Drug Targets Topological Structure Characteristics

According to analysis on intermediary and source functions of the drug targets, there are few significant characteristics with respect to targets discovery. These results indicate that the function of spreading drug stimulus seems not to be as important as the traditional view for the drug targets. Actually, several studies show that drug effects only depend on some special proteins rather than impacting most of proteins. For a specific drug, the physical and chemical properties of the target proteins are exactly important for response on the drug stimulus. Moreover, these proteins with different physical and chemical properties often have different topological features.

The process of finding a new drug against a chosen target for a particular disease usually involves high-throughput screening (HTS), wherein large libraries of chemicals are tested for their ability to modify the target [51]. For example, if the target is a novel GPCR, compounds will be screened for their ability to inhibit or stimulate that receptor (see antagonist and agonist): if the target is a protein kinase, the chemicals properties will be tested for their ability to inhibit that kinase. The physicochemical properties associated with drug absorption include ionization (pKa) and solubility; permeability can be determined by PAMPA and Caco-2. PAMPA is attractive as an early screen due to the low consumption of drug and the low cost compared to tests such as Caco-2, gastrointestinal tract (GIT), and Blood-brain barrier (BBB) with which there is a high correlation. Another important method for drug discovery is drug design, whereby the biological and physical properties of the target are studied, and a prediction is made of the sorts of chemicals that might, for example, fit into an active site. One example is fragment-based lead discovery (FBLD) [52]. Novel pharmacophores can emerge very rapidly from these exercises. In general, computer-aided drug design is often but not always used to try to improve the potency and properties of new drug leads.

Meanwhile, eigenvector centrality also implied that the drug targets may have the special structure that causes their roles on connectivity to be equal to or even more important than other proteins with higher degree and betweenness. Some structures are found to link with significant proportion of proteins. In the yeast proteins interaction networks, the protein pairs inside some special subnetworks corresponding to protein complexes tend to show interactions maps of specific biological processes [53]. In breast cancer prognosis, the changes of network modularity may be a defining feature of tumor phenotype that determines patient prognosis [54]. Moreover, the average clustering coefficient values of the cancer proteins interaction networks were lower. It implies that the proteins have a lower tendency to form clusters [42]. Similarly, the clustering coefficient of the drug targets (0.06) is lower than other proteins (0.12) in drug proteins interaction networks as well. Furthermore, most drug targets distribute during 0 to 0.1. As eigenvector centrality, it implies the drug targets may be in some special subnetworks. In this section, beside the physical and chemical properties, we examined which topological characteristics of the PPI networks contribute for response to the drug stimulus based on modularity and coreness.

#### 3.3.1. Modularity

Modularity is the degree to which the components of the networks may be separated and recombined. The definition of modularity varies in different fields with similar essence. In biology, modularity refers to the concept that organisms or metabolic pathways are composed of modules. In the paper, we studied modularity *Q* of networks, which is a benefit function defined as (4) that measures the quality of a division of a network into groups or communities [55].

(4) $$\begin{array}{c} Q=\frac{1}{2m}\underset{i,j}{\sum }\left(\;A_{i,j}-\frac{k_{i}k_{j}}{2m}\;\right)\delta \left(\;c_{i},c_{j}\;\right), \end{array}$$

where the degree of node *i* assigned to community *c*_*i*_ is *k*_*i*_, if there is no interaction between proteins *i* and *j*, and 1 otherwise, *m* = (1/2)Σ_*i*,*j*_*A*_*i*,*j*_, and *δ*(*c*_*i*_, *c*_*j*_) is 1 if *c*_*i*_ = *c*_*j*_ and 0 otherwise.

**Figure 6:**
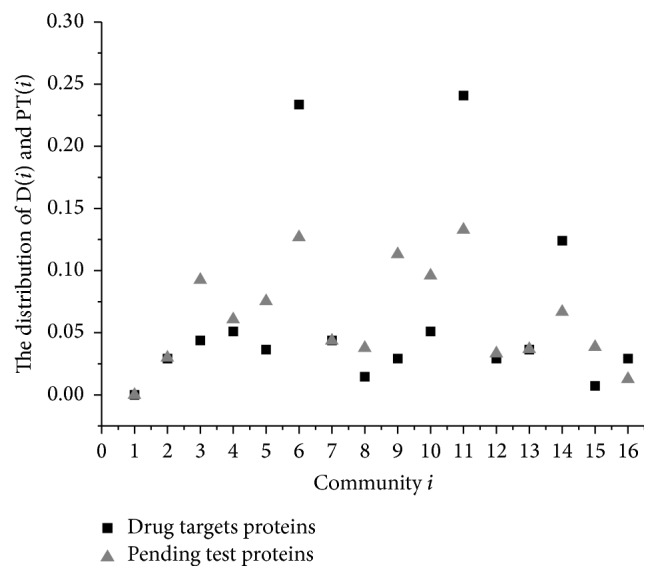
The distribution D(*i*) and PT(*i*) of the communities. There are 15 main protein communities consisting of 11,099 proteins detected in PPI. The majority of the drug targets are in the three communities 5, 10, and 13.

There are many effective algorithms to detect communities by maximizing the modularity based on (4). In the paper, we used the method in paper [56] and detected 34 communities. There are 15 main protein communities consisting of 11,099 proteins detected in PPI. The distributions D(*i*) and PT(*i*) of the communities are shown in Figure 6, where D(*i*) is the percentage of the drug targets in community *i* to the amount of known drug targets. Similarly, PT(*i*) is the percentage of the pending test proteins detected into community *i*.

However, if a community has most of the proteins, it also probably contains most of the drug targets. Such community should not be considered as a target-like community which mainly consists of drug targets. Furthermore, we calculate rd(*i*) = D(*i*)/PT(*i*) as the degree to which community *i* is likely to be a target-like community. Because D(*i*) = *i*_D_/|D| and PT(*i*) = *i*_PT_/|PT|, rd(*i*) = (D(*i*)/PT(*i*))/(|D|/|PT|), where *i*_D_ and *i*_PT_ are the amounts of the drug targets and other proteins in community *i*, respectively, and |D| and |PT| are the amounts of the set D and of the set PT.

Therefore, rd(*i*) > 1 indicates that *i*_D_/*i*_PT_ > |D|/|PT|. In other words, community *i* tends to be as a target-like community. Similarly, if rd(*i*) < 1, community *i* is closer to nondrug target proteins.

Figure 6 shows 15 communities which contain the most drug targets. In particular, the known drug target proteins mainly gathered around communities 5, 10, and 13. These three communities account for almost 66% known drug targets. In contrast, the pending test protein is flatter and more spread out for several communities. It implies there are less nontarget data around the communities in which drug target data mainly clustered together. In particular, Figure 8 shows communities 5 and 10 in which the drug target proteins most mainly distribute. In Figure 8, the drug target is not always the one with highest degrees in either whole protein network or communities they mainly cluster.

**Figure 7:**
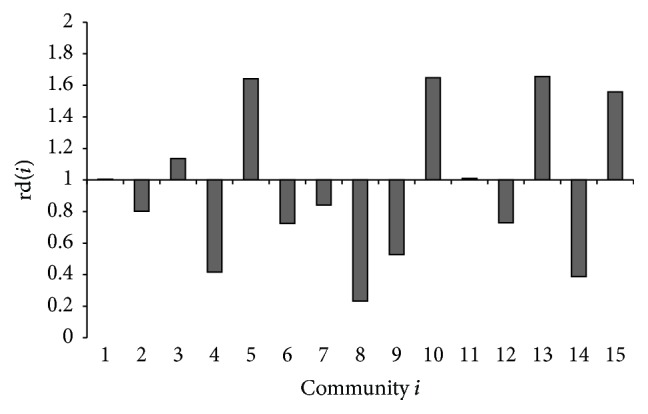
rd(*i*) of the communities. When rd(*i*) > 1, it indicates that the community *i* tends to be a target-like community. Similarly, if rd(*i*) < 1, the community *i* is closer to nondrug target proteins.

**Figure 8:**
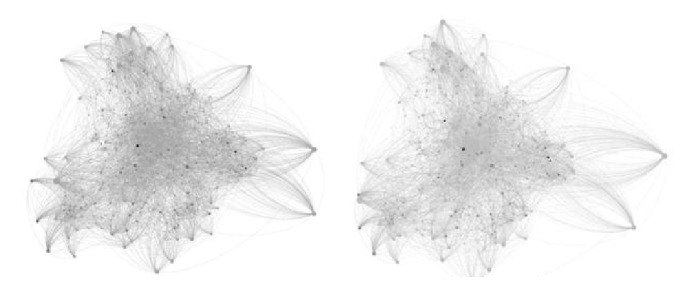
The full view of communities 5 and 10.

These three communities also have highest rd(*i*) in Figure 7. In addition, community 15 is the other significant target-like community as well. Meanwhile, the nondrug targets are clustered in communities 4, 8, 9, and 14. The results based on modularity imply there are target-like communities existing in the PPI networks. It is helpful to study which proteins are the potential drug targets and to understand how the drug targets work. Actually, some approaches have been explored for drug discovery by specific structural and physicochemical properties to ensure efficacious, bioavailability, and safety. They propose the concept of druggable proteins to bind potentially effective drug-like small molecules. One example is to identify metabolic enzymes as drug targets by searching for similar structural properties of known drug targets in other organisms [57].

#### 3.3.2. Coreness

A *k*-core of a graph *G* is a maximal connected subgraph of *G* in which all vertices have degree *k* at least. There is a way to determine a *k*-core by iteratively pruning nodes with a degree lower than *k* and their incident links [58]. The coreness of a protein is *n* if this protein is in the *n*-core of the PPI network but not in the *n* + 1-core. We examined rc_D_(*k*) and rc_PT_(*k*) defined as follows for drug targets and other proteins in *k*-core, respectively.

(5) rcDk=pkDDRrcPTk=pkPTPR,

where *p*_*k*_^D^ and *p*_*k*_^PT^ denote the proportion of the drug targets and other proteins in *k*-core of the PPI network, respectively. DR and PR are the drug target's ratio and pending test ratio in Table 1, respectively.

As rd(*i*) is used in analysis of the modularity above, rc_D_(*k*) > 1 indicates that the drug target proportion is higher than global level. In other words, the *k*-core tends to be a target-dominated subnetwork. In turn, if rc_D_(*k*) < 1, the *k*-core mainly consists of nondrug target proteins.  Figure 10 shows rc_D_(*k*) and rc_PT_(*k*) of the drug targets PPI networks.

Coreness poses a systematic way to consider the local and global significance of a protein, which indicates the inherent layer structure of the PPI network. Its biological significance has been found in several studies. For example, the functions of some function-unknown proteins of* E. coli* are predicted based on coreness [59]. The probability of yeast proteins both being essential and evolutionary conserved successively increases toward the innermost cores [60]. Disease-resistant domains have higher coreness than other and disease-susceptible domains [61]. In our study, the coreness shows significant difference between drug targets and normal proteins (see Figure 10). The drug target proteins are mainly in 6, 9, 12, 16, and 18 cores. In contrast, other proteins are evenly distributed over all the possible cores.

**Figure 9:**
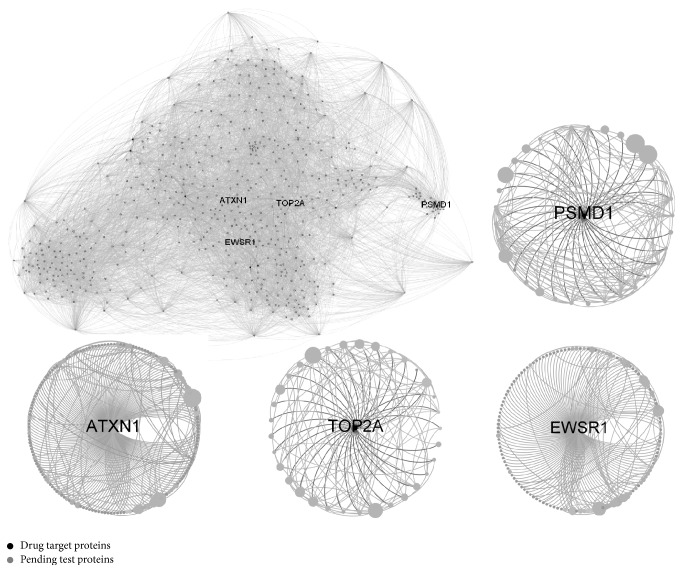
17-core subnetwork of the drug targets PPI network. As shown in four ego networks of the known drug targets (PSMD1, TOP2A) and normal proteins (EWSR1, ATXN1), the neighbors of a drug target are less but those neighbors have higher degree than normal proteins. It implies a very important drug protein's reaction mechanism, that is, the drug target protein's interaction with most high connective proteins, though few of them are the hubs as important bridges of the PPI network.

**Figure 10:**
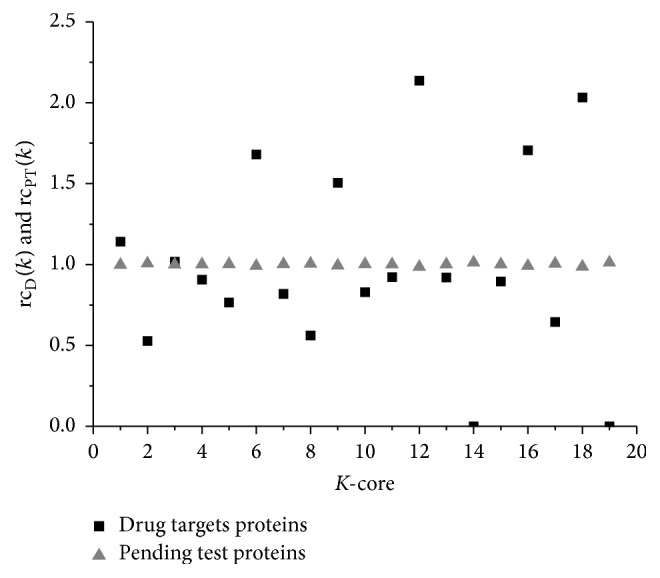
rc_D_(*k*) and rc_PT_(*k*) of the drug targets PPI networks. The drug target proteins are mainly in the 6-, 9-, 12-, 16-, and 18-core subnetworks, while the pending test proteins that represent normal proteins are evenly distributed across all core subnetworks.

The proteins in the innermost *k*-cores, which are not necessarily among the highest connected ones, can interact with most high connection proteins [60]. As shown in Section 3.1, eigenvector centrality of the drug targets is not lower than normal proteins even though the known drug targets have lower degree and betweenness. For example, degrees of the drug targets PSMD1 and TOP2A are 33 which are less than normal proteins EWSR1 (135) and ATXN1 (171). But they are all in the 17 cores of the PPI network (see Figure 9). Moreover, the coreness of PSMD1 and TOP2A is 18 while coreness of EWSR1 (135) and ATXN1 is 17. Figure 9 shows the neighbors of the drug targets PSMD1 and TOP2A are less but have higher degree than normal proteins EWSR1 and ATXN1.

More generally speaking, coreness and eigenvector imply that the drug target proteins may not be the hub in the PPI network but they are able to propagate the affection to some hub-like proteins and spread the transcription signal to other related proteins through them. One of the possible reasons is that, in the cancer network, for example, only a part of the interactions among the related proteins may be active at a specific condition [62]. Therefore, the original hub is very likely to change into a normal protein. On the other hand, although the drug signal may be spread widely through hubs to inhibit the disease function, it is almost inevitable that it becomes an obstacle for many essential functions. But if drug stimulus is diffused indirectly, the drug signal may be more suitable for the tradeoff. According to the analysis about drug target structure characteristics, although the drug targets have few significant functions as intermediary and source of the drug, it is possible to identify potential drug targets based on their special structure characteristics.

## 4. Discussion

To understand the mechanism of drug targets at the molecular level where most drugs work, we studied the topological characteristics of the drug targets with three plausible views from which a drug target is known as (1) the intermediaries which play an important role on interactions of the drug targets network; (2) the sources which convert drug stimulus into the desirable therapeutic effects and spread to other proteins; (3) the proteins which have special topological and functional significance. From a series of their topological indices, we found somewhat surprising conclusions.

One conclusion was that known drug targets do not have the privilege of the first two roles although the drug fulfills its function by interactions with a target protein. In other words, the function of spreading drug stimulus seems not to be important for the drug targets. Meanwhile the drug targets have special topological structures that are different with normal proteins. We suspect that these special topological structures may help drug targets to respond to drug stimulus. Actually, the fact has been shown in some studies. For example, Overington et al. [4] suggested that most drugs depend on multiple specific motifs of the PPI networks. Hopkins [63] reported that it is necessary to map drug targets into integrated biological networks to identify the optimal points of protein-protein interactions for drug discovery. Ma'ayan et al. [64] argued that several classes of proteins with some special network statistics in the human genome appear to be better targets for drugs. Nacher and Schwartz [65] found that drugs usually have a high centrality value in the drug-therapy network and act on multiple molecular targets in the human system. Sakharkar et al. [66] showed that proteins with single-exon gene architecture are more likely to be targetable.

As the toy example shows the effectiveness of the features we discussed in the paper, we just use some traditional methods to detect drug targets with the features we discussed. These experiments concern two cases. One is to use all features including degree, betweenness, eigenvector centrality, average distance, eccentricity, modularity, clustering coefficient, community, and coreness, and the other is to use the drug target proteins' particular features. These three features were eccentricity, modularity, and coreness. This shows that the accuracy of the classifiers can benefit from using these particular features no matter what methods used to build classifier. All of the algorithms were performed on the original collected dataset with the help of Weka [67]. The details on results can be found in Table 2.

Although the causes are unknown currently, it is very helpful for drug discovery by reducing potential drug target proteins. Because at present the chemical and physical properties of known drug targets can be found but nondrug targets cannot, the prediction of drug targets is one classification problem for which there is no good solution. Currently, most studies usually use pending test proteins as nondrug targets, but they inevitably contain proteins that subsequently turn out to be drug targets although the majority of the proteins are normal proteins [28]. Therefore, there is an alternatively reasonable way to collect a more accurate nondrug targets dataset based on the drug targets structure characteristics.

Here we use modularity and coreness by naive Bayes to select nondrug targets from PT1 in which every protein consists of 39 chemical and physical properties. Assume that each protein has a 50% probability as a drug target in the pending test dataset. From 1,180 proteins in PT1, we selected 1,180*∗*0.5 = 590 proteins as the nondrug target dataset by the probabilities calculated by naive Bayes. By using the drug target dataset (D) and nondrug target dataset, we trained the drug target classifier based on support vector machines (SVMs) by Weka [67]. Finally, 102 proteins are predicted as potential drug target proteins from PT1. The accuracy of the classifier is 82.01% by 10-fold cross-validation. Positive predictive value is 72.7% and negative predictive value is 82.4%. These results indicated that SVMs could be reasonable in the prediction of drug target proteins. Particularly, some of the prediction results were found as published new drug targets recently. For example, CTBP1 is listed by Cancer Resource [68], and TASP1 is also a drug target, which is used to combat novel diseases whose candidate genes are targeted by HNF4alpha splice variants in hepatocellular carcinomas [69], as well as PNKP and RAG2 [70].

## 5. Conclusion

Protein-protein interactions are a better way to understand the biological functions through systemic view, which have been widely examined in many research studies under the molecular level. In this paper, the contrastive analysis on the topology of PPI networks between drug target proteins and other proteins provided a systemic biological mechanism of drug target protein interactions.

We found that 5 topological indices (degree, betweenness, eigenvector centrality, average distance, and cluster coefficient) are quite similar between drug target proteins and other proteins in the PPI network. It implies that known drug target proteins do not have the privilege of being a drug effect intermediary and/or source. It is different with some traditional views that intuitively consider a drug target protein as a hub of the PPI network.

On the other hand, a drug target protein has its own particular characteristics on the other 3 topological features including eccentricity, modularity, and coreness. It implies a drug target protein may have the capability to interact with some hub proteins, which pass their biological stimulus to other related proteins. These topological characteristics are helpful to understand how the drug target proteins work and test new drug effects. For example, one of the usages is to identify the new drug target. At present, there is no nondrug target dataset like the drug target dataset that records chemical and physical properties. However, according to these topological characteristics, the nondrug target proteins can be gathered. Hence, prediction of drug target proteins becomes a classic data mining problem which can be solved by many supervised learning algorithms. In the paper, 102 potential drug target proteins are predicted by SVMs, some of these have been published recently. It is just a simple example. One of the further works is to improve the prediction by examining these chemical and physical properties combined with topology. On the other hand, although there are improved methods to reduce the protein-protein interactions false positive rate [32, 71], the protein-protein interactions network always has a fairly high false positive rate. New high-throughput data and PPI database updates are important in the future.

## Figures and Tables

**Table 1:** Summary of data.

	Drug targets	Pending test (PT)		Proteins	Edges	Drug targets ratio	Pending test ratio
	(D)	PT1	PT2	(P)	(E)	(DR = D/P)	(PR = PT/P)
Original data	149	1212	10197	11558	65772	0.23%	99.77%
Used data	138	1180	9983	11301	65547	0.21%	99.79
Coverage ratio	92.6%	97.4%	97.9%	97.8%	99.7%	91.3%	100%

**Table 2:** The results of the experiments (10-fold cross-validation).

Algorithm	Using all features (8 topological features)			Using particular characteristics (3 topological features)		
	Accuracy	Positive predictive value	Negative predictive value	Accuracy	Positive predictive value	Negative predictive value
C4.5	65.2%	63.4%	66.5%	68.7%	67.7%	69.5%
Logistic regression	54.2%	52.8%	56.0%	67.6%	63.4%	68.0%
Naive Bayes	56.8%	54.4%	58.2%	67.3%	64.9%	68.0%
Bayes network	64.5%	63.4%	65.0%	70.6%	67.3%	71.5%
SVM	65.8%	60.2%	67.4%	72.0%	69.7%	73.4%
Radom forest	63.2%	60.4%	65.5%	69.6%	66.4%	70.5%
